# In vivo and molecular docking investigation of the novel anti-inflammatory and mitochondrial mechanisms underlying the renoprotective effects of resmetirom and curcumin in gentamicin-induced renal toxicity in rats

**DOI:** 10.1186/s12882-026-04928-8

**Published:** 2026-04-13

**Authors:** Amira S. Ahmed, Mahmoud S. Sabra, Eman S. Shaltout, Zainab S. Abdelqader

**Affiliations:** 1 Histology Department, Faculty of Medicine, Assiut University, Assiut, 71526 Egypt; 2 Pharmacology Department, Faculty of Veterinary Medicine, Assiut University, Assiut, 71516 Egypt; 3 Forensic Medicine and Clinical Toxicology Department, Faculty of Medicine, Assiut University, Assiut, 71526 Egypt

**Keywords:** Gentamicin, Resmetirom, Curcumin, Nephrotoxicity, Oxidative stress, Mitochondrial dysfunction, Molecular docking

## Abstract

**Background:**

Gentamicin (GS) is a potent aminoglycoside antibiotic whose clinical use is limited by nephrotoxicity associated with oxidative stress, mitochondrial dysfunction, and inflammation. This study aimed to explore the renoprotective mechanisms of resmetirom (RES) and curcumin (CUR) against GS-induced renal injury in rats, emphasizing anti-inflammatory and mitochondrial pathways and supporting findings through molecular docking.

**Methods:**

Twenty-four adult male Wistar rats were randomly allocated into four groups (*n* = 6): control, GS (100 mg/kg, i.p.), GS + CUR (200 mg/kg, oral), and GS + RES (3 mg/kg, oral). Treatments were administered for seven days. Serum renal biomarkers (urea, creatinine) and tissue oxidative stress markers (MDA, NO, GSH) were quantified using spectrophotometric methods. Specific kidney damage indicators for kidney injury molecule-1 (KIM-1) and neutrophil gelatinase-associated lipocalin (NGAL) were evaluated. Gene expressions of dynamin-related protein 1 (DRP1) and AKT Serine/Threonine Kinase 1 (AKT1) were analyzed by quantitative RT-PCR, while mammalian target of rapamycin (mTOR) and forkhead box protein O1 (FOXO1) protein expression were assessed immunohistochemically. Histopathological and ultrastructural evaluations were conducted using light and electron microscopy. Molecular docking was performed using AutoDock Vina to assess binding affinities of CUR and RES with AKT1, DRP1, FOXO1, and mTOR.

**Results:**

GS administration significantly elevated serum urea and creatinine and increased renal MDA levels while decreasing NO and GSH. These alterations were markedly attenuated by CUR and RES, with RES showing superior improvement in renal function and oxidative balance. qPCR analysis revealed significant GS-induced upregulation of AKT1 and DRP1, which was normalized by both CUR and RES. Immunohistochemistry demonstrated downregulation of mTOR and FOXO1 following GS exposure, with substantial restoration after CUR and RES treatments. Histopathological and ultrastructural analyses confirmed the protective effects of both compounds, showing preserved glomerular and tubular integrity.

**Conclusion:**

Resmetirom and CUR showed significant renoprotective benefits against GS-induced nephrotoxicity by alleviating oxidative stress, modulating mitochondrial dynamics, and restoring cellular signaling pathways involving AKT1, DRP1, mTOR, and FOXO1. The molecular docking results corroborate their direct interactions with these targets, highlighting RES as a promising therapeutic agent for nephroprotection.

## Introduction

Drug-induced nephrotoxicity is a major contributor to acute kidney injury in hospitals, responsible for up to 60% of cases in certain patient demographics, and is associated with elevated morbidity and mortality rates in both children and adults [1]. Antibiotics, including vancomycin, cephalosporins, and aminoglycosides, are prevalent causes of drug-induced nephrotoxicity, alongside other pharmaceuticals like NSAIDs and chemotherapeutic agents [2, 3]. The mechanisms of damage are diverse and may encompass direct tubular injury, inflammation (acute interstitial nephritis), or tubular blockage [4].

Gentamicin (GS) is a bactericidal, broad-spectrum antibiotic, ineffective against streptococci and anaerobic bacteria. The mechanism of action entails the suppression of bacterial protein synthesis through binding to 30S ribosomes [5]. The principal adverse effect of GS is nephrotoxicity. 10% to 25% of those subjected to GS develop acute renal damage. Due to its efficacy and low cost, GS continues to be utilized despite its detrimental effects [6]. A regimen of intraperitoneal administration of 100 mg/kg body weight to rats once daily for seven consecutive days resulted in acute renal failure. The accumulation of GS in the renal cortex causes nephrotoxicity through oxidative stress and tubular necrosis [3].

The precise mechanisms through which GS may induce nephrotoxicity remain unidentified; however, mitochondrial damage and disruption of energy production, resulting in the generation of reactive oxygen species (free radicals), are regarded as the principal mechanisms of aminoglycoside-induced renal injury, culminating in cellular apoptosis [7, 8]. Furthermore, a complex cascade involving oxidative damage, inflammation, and cellular apoptosis is implicated in GS-induced renal failure. Phospholipidosis, inflammation, necrosis, oxidative stress, and GS binding to phospholipids all contribute to membrane integrity being compromised [9]. The production of reactive oxygen and nitrogen species by the mitochondria in the renal cortex causes cell death and increases levels of pro-inflammatory cytokines and nuclear factor kappa-b [10].

Gentamicin recently reported promoting apoptosis via the Bax/Bcl2-Caspase3 pathway, altering the expression of renal transporters, impairing mitochondrial homeostatic balance, and altering the expression of mitochondrial dynamics (e.g., Optic atrophy 1, Mitofusin1/2, and DRP1). These effects worsened renal damage [11]. Mitochondria are both targets and amplifiers of cellular toxicity; furthermore, GS is known to directly affect mitochondrial ribosomes and gene expression. Antioxidants and mitochondrial transplantation are two examples of therapeutic treatments that target mitochondrial integrity and may provide nephroprotection [12].

Curcumin (CUR), a bioactive polyphenol, has been thoroughly investigated for its pharmacological attributes, encompassing antioxidant, anti-inflammatory, and possible anticancer activities [13]. Its capacity to neutralize free radicals, inhibit nuclear factor kappa-light-chain- enhancement of activated B cell signaling, and alter apoptotic pathways amplifies its renoprotective efficacy [14]. Recently, CUR mitigated acute kidney injury, presumably by inhibiting inflammation and ferroptosis through the long-chain acyl-coenzyme synthetases 4/glutathione peroxidase 4 signaling pathway [15]. CUR is widely acknowledged as safe, endorsing its application as a therapeutic agent in renal injury induced by oxidative stress [14].

Resmetirom (RES), a selective agonist of the thyroid hormone receptor beta, predominantly affects the liver to diminish oxidative stress, inflammation, and steatosis while alleviating fibrosis [16]. In addition to hepatic effects, thyroid hormone receptor beta is present in renal tissue, and preclinical studies indicate that its activation may provide renoprotective benefits by reducing oxidative damage and inflammatory responses [17, 18]. These characteristics position RES as a potential adjuvant in preventing or mitigating GS-induced nephrotoxicity.

Resmetirom and CUR provide a multi-pronged strategy for improving patient outcomes in acute renal injury treatment by incorporating complementary and alternative medicine techniques. There is an increasing demand for nephroprotective measures and dependable diagnostic indicators [19]. Both the kidneys and the liver have high concentrations of thyroid hormone beta receptors, and there are many shared pathophysiological features and risk factors between chronic kidney disease and nonalcoholic fatty liver disease. Consequently, beta receptor agonists for thyroid hormones show promise as a treatment for chronic renal disease [20].

In the past decade, research has shown that metabolic-associated fatty liver disease can affect liver prognosis and also serve as a risk factor for various chronic diseases, including cardiovascular disease, chronic kidney disease, and cancers outside of the liver [21]. March 2024 saw the FDA’s approval of the new drug RES for the treatment of steatohepatitis in patients with non-cirrhotic metabolic dysfunction [22]. RES shows promise in reducing GS-induced kidney damage due to its anti-inflammatory, antifibrotic, and antioxidant characteristics.

The present study utilized in *silico* molecular docking to investigate the binding affinities of CUR and RES with critical signaling proteins implicated in mitochondrial regulation and inflammatory pathways, specifically AKT1, FOXO1, DRP1, and mTOR. This integrative experimental-computational approach sought to clarify the mechanisms by which these drugs may alleviate gentamicin-induced nephrotoxicity, notably through the control of oxidative stress, inflammatory responses, and mitochondrial dynamics. Furthermore, evaluate the renoprotective effects of CUR and RES on biochemical and histological markers in a GS-induced nephrotoxic model.

## Materials and methods

### Design of experimental animals

The 24-adult male Wistar albino rats used in this study were recruited from the Experimental Animal Facility at Assiut University. The rats ranged in weight from 150 to 200 g and were 6 to 10 months old. All of the animals were kept in typical laboratory cages with a 12-hour light/dark cycle and kept at a constant room temperature. All the while the trial was underway, the rats were given free access to a normal rodent chow diet. With approval number 06/2025/362, the Veterinary Medicine Institutional Animal Care and Use Committee at Assiut University has given the go-ahead for all experimental operations that adhere to the National Institutes of Health’s regulations for the treatment and care of research animals.

Every day for seven consecutive days, the following treatments were administered to the animals that had been randomly divided into four equal groups, with six animals in each group. I. Control Group: 0.5 ml of isotonic saline was injected intraperitoneally (i.p.). Group II, which was administered GS (Memphis for Pharmaceuticals & Chemical Industries, Egypt intraperitoneally once daily at a dosage of 100 mg/kg, in accordance with the Yaman and Balikci [23] procedure. Group III (GS + CUR) Animals were given CUR (Cat. no. 2340AP, AKSCI, USA) in an isotonic saline suspension orally at a dosage of 200 mg/kg/day [24]. Then, 100 mg/kg of GS was injected intraperitoneally one hour later. Suspended in isotonic saline, RES (Cat. No. 2544AH, AKSCI, USA) was administered to Group IV (GS + RES). According to Caddeo, Serra [25], the dosage that was given daily in the form of water was 3 mg/kg. Thereafter, 100 mg/kg of GS was injected intraperitoneally once for the next hour.

### Collection of samples

Under light anesthesia, blood samples were extracted from the retro-orbital plexus utilizing a glass capillary tube at the conclusion of the investigation, allowed to coagulate at ambient temperature, and subsequently centrifuged at 700×g for 20 minutes (Thermo Fisher Scientific, USA). The serum was isolated and preserved at −80 °C for subsequent examination. All groups of rats were euthanized using 5% isoflurane anesthesia as a technique of euthanasia. Upon the rats’ failure to respond to stimulation of the head and limbs, we promptly euthanized them by dislocating their necks. Rats were considered deceased ten seconds post-cervical dislocation if they ceased respiration and exhibited no response to systemic stimulation [3]. The kidneys were excised, rinsed with ice-cold isotonic saline, dried using a blotting apparatus, and subsequently placed into a tissue homogenizer (Model T25 digital ULTRA-TURRAX®, Germany) for weighing, division, and homogenization in ice-cold phosphate-buffered saline (PBS, pH 7.4). Following centrifugation of the homogenates at 10,000 rpm for 20 minutes at 4 °C, the residual liquid was frozen at −80 °C for further biochemical analysis. Kidneys from all experimental groups were meticulously removed, preserved in the suitable fixative, and analysed using histopathological, immunohistochemical, and ultrastructural (electron microscopy) methods [26].

### Molecular docking of protein-ligand complexes

The methodologies delineated by El Tabaa, Faheem [27] were adhered to for executing the molecular docking techniques. A molecular docking protocol was employed, incorporating the three-dimensional conformations of CUR (https://pubchem.ncbi.nlm.nih.gov/compound/Curcumin) and RES (https://pubchem.ncbi.nlm.nih.gov/compound/Resmetirom), alongside the protein structures of AKT1, DRP1, FOXO1, and mTOR.

To perform molecular docking with the Autodock Vina protocol, each file containing CUR and RES molecules together with the protein targets was individually imported into PyRx (version 0.8) [28]. The binding energies of FDA-approved pharmaceuticals varied from −5.63 to −6.85 kcal/mol [29]. The final phase involved utilizing BIOVIA Discovery Studio Visualizer 2024 to illustrate protein-ligand interactions [30].

### Renal function biomarkers

The serum urea concentration was measured using a colorimetric assay kit (Spectrum Diagnostics, Egypt; REF: 318 001), employing the urease–salicylate–hypochlorite enzymatic approach, a variant of the traditional urease–Berthelot reaction. The serum creatinine concentration was quantified via the Jaffé kinetic method with a colorimetric test kit (Spectrum Diagnostics, Egypt; REF: 318 002), in accordance with the manufacturer’s guidelines [31].

### Oxidative stress biomarkers

Nitric oxide (Cat. no. E-BC-K035-M), GSH (Cat. no. E-BC-K030-S), and MDA (Cat. no. E-BC-K025-S) concentrations in kidney tissue homogenates were assessed spectrophotometrically using a commercially available kit from Elabscience in China [32].

### Kidney injury markers assessment

We used a rat-specific sandwich ELISA kit from Sunlong Biotechnology in Shanghai, Hangzhou, Zhejiang, China, to measure the levels of KIM-1 (Cat. no. E-EL-R3019) and NGAL (Cat. no. E-EL-R3055) in kidney tissue [31].

### Extraction of RNA and Quantitative real-time polymerase chain reaction (qPCR) for DRP1 and AKT1

The experimental animals’ frozen kidney tissues were processed to isolate total RNA using the RNeasy Mini Kit (Cat. no. 74106, Qiagen, Germany) following the manufacturer’s protocol. The extracted RNA (500 ng) was used to synthesize complementary DNA using the High-Capacity cDNA Reverse Transcription kit with RNase inhibitor (Cat. no. 4374967, Thermo-Fisher Scientific, USA) after quantification with a nanodrop spectrophotometer (Epoch Microplate Spectrophotometer, Biotech, VA, USA). Following that, the cDNA was used as a template for the target genes DRP1 and AKT1 and was amplified using the Maxima SYBR Green qPCR Master Mix kit (Cat. no. K0222, Thermo-Fisher Scientific, USA). Primer sets are utilized for amplification as shown in Table 1. We used the 2 − ΔΔCT method to standardize the data against the control β-actin and display it as fold changes in mRNA expression [33].

**Table 1:** Primers for real-time polymerase chain reaction

Genes	5′−3′ primer sequence	Accession number
DRP1	**Forward:** AGAAAAGGAAGCAAGCGGGC **Reverse:** TGACAACGTTGGGCGAGAAA	**NM_001437688.1**
ACT1	**Forward:** GTGGCAAGATGTGTATGAG **Reverse:** CTGGCTGAGTAGGAGAAC	**XM_039111773.2**
Beta actin	**Forward:** CACTATCGGCAATGAGCGGTTCC **Reverse:** CAGCACTGTGTTGGCATAGAGGTC	**NM_031144.3**

Dynamin-related protein 1 (DRP1), AKT Serine/Threonine Kinase 1 (AKT1)

### Histopathological and immunohistochemical examination

The kidney tissues were fixed in 10% formalin, underwent processing, and were subsequently embedded in paraffin as formalin-fixed paraffin-embedded tissue sections. The general histological architecture was evaluated under light microscopy by cutting and staining sections with hematoxylin and eosin (H&E) that were 5–7 μm thick [34].

Mounted on slides coated with poly-L-lysine, paraffin-embedded kidney slices were deparaffinized in xylene and rehydrated using a gradient of alcohols leading to distilled water. To accomplish antigen retrieval, the sections were heated for 10 minutes in a 10 µM citrate buffer with a pH of 6.0. A mouse monoclonal antibody against mTOR (Cat. No. 215Q18) and a recombinant rabbit monoclonal antibody against FOXO1 (Cat. No. MA5-32114) were used as primary antibodies for the subsequent incubation of the sections. Thermo Scientific, USA, was the source for both antibodies. One way to identify it was using the avidin-biotin-peroxidase complex technique. The chromogen was hydrogen peroxide, and the color developer was 3,3′-diaminobenzidine. The sections were subsequently dried, cleaned, and stained with Mayer’s hematoxylin for counterstaining [35, 36]. For the negative control sections, phosphate-buffered saline was used as the incubation medium rather than the primary antibody. The antibody source suggested using testis for FOXO1 and brain for mTOR as positive control tissues. Cells that tested positive for the antibody showed brown staining in the cytoplasm and blue counterstaining in the nucleus.

As per earlier research, the total histological scores, as well as those for tubular degeneration, necrosis, and tubule interstitial nephritis, might take on values between zero and four [3].

### Electron microscopic examination

A minimum of 24 hours was required to fix the kidney tissues that were to be used for ultrastructural examination in 5% glutaraldehyde. The tissues were prepared into semi-thin slices (0.5–1 μm) and dyed with toluidine blue in order to produce representative areas. These slices were subsequently examined under a light microscope. After slicing them into tiny sections of 50–80 nm, we mounted them on copper grids and dyed them with uranyl acetate and lead citrate [37]. Sections were examined and photographed using a transmission electron microscope manufactured by JEOL (Tokyo, Japan) with an operating voltage of 80 kV at the Electron Microscope Unit, Assiut University.

### Morphometric analysis

Tissue sections were photographed using a computer-assisted image analyzer (Soft Imaging System, Olympus, Japan) at the Department of Histology, Faculty of Medicine, Assiut University. The number of positive FOXO1 and mTOR immunostained cells was quantified using ImageJ software (NIH, USA) from 10 randomly selected, non-overlapping high-power fields (×400) per animal in each group [38].

### Statistical analysis

The Shapiro-Wilk test was used to ensure that all parameters we looked at followed a normal distribution. We used one-way or two-way analysis of variance (ANOVA) followed by Tukey’s post hoc test. We defined statistical significance at *p* ≤ 0.05. The results are shown as the mean ± standard error (SE). We used GraphPad Prism® software (Version 10.6.0; GraphPad Software Inc., San Diego, CA, USA) for all analysis.

## Results

### Docking study on the relationship between resmitrom and curcumin and important targets

**Fig. 1:**
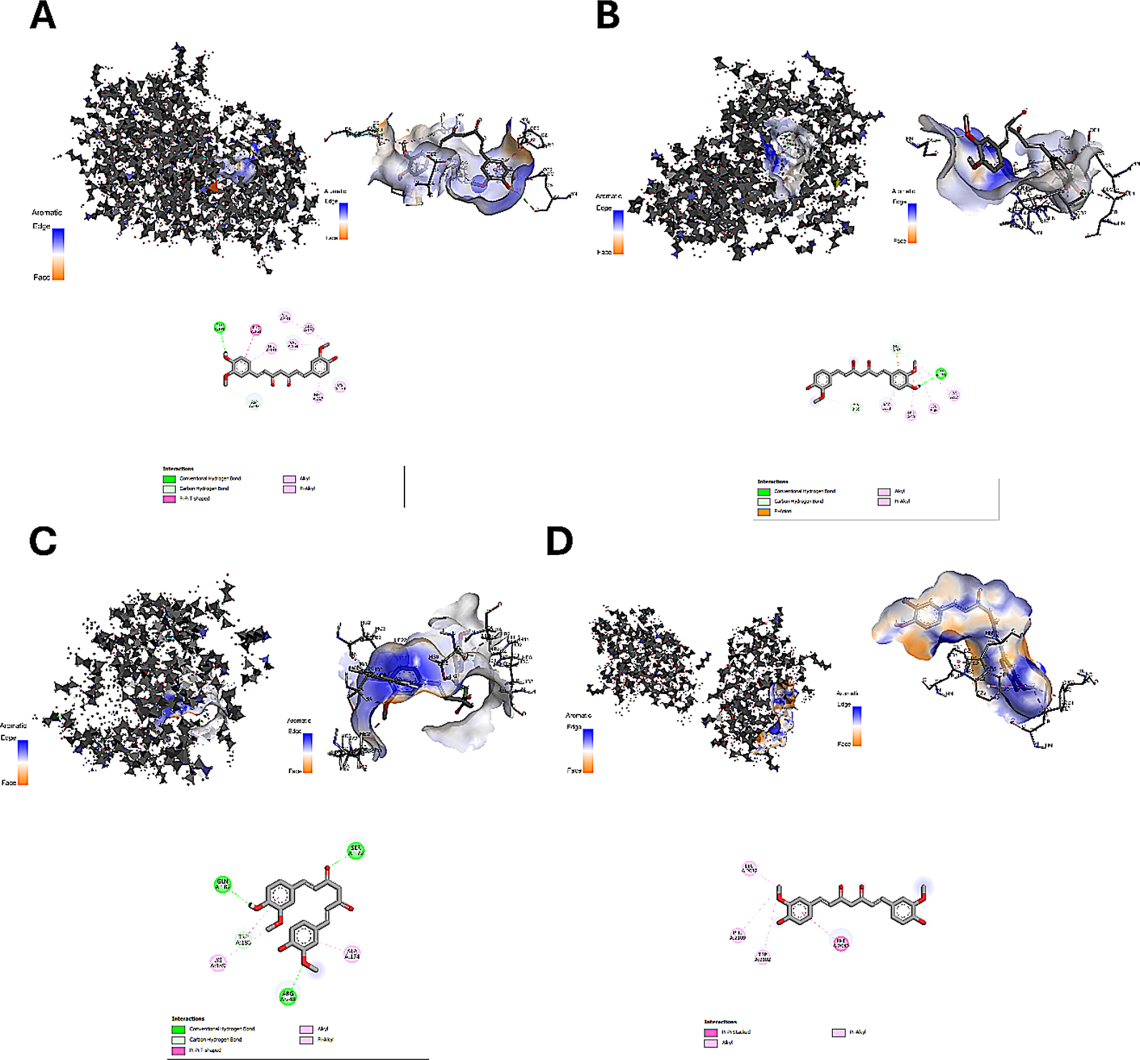
Molecular docking results of curcumin with (**A**) AKT1 (PBD ID: 6ccy), (**B**) DRP1 (PBD ID: 5ons), (**C**) FOXO1 (PBD ID: 6qvw), and (**D**) mTOR (PBD ID: 1aue). AKT Serine/Threonine kinase 1 (AKT1), Dynamin-related protein 1 (DRP1), Forkhead box protein O1 (FOXO1), and Mammalian target of rapamycin (mTOR)

The CUR and RES compounds were confirmed to be associated with AKT1, DRP1, FOXO1, and mTOR targets by a docking analysis. The interactions between CUR and AKT1, DRP1, FOXO1, and mTOR had the highest binding affinities (−7.3, −6.3, −6.1, and −8.5 kcal/mol, respectively), according to the molecular docking results (Table 2; Fig. 1).

**Table 2:** Molecular docking procedure involving the curcumin molecule and key targets

Compound name	Targets	Gene name	PDB number		Binding AFFINITIES (kcal/mol)
CuRcumin (cur)	AKT Serine/Threonine Kinase 1	AKT1	6ccy		−7.3
	Dynamin-related protein 1	DRP1	5ons		−6.3
	Forkhead box protein O1	FOXO1	6qvw		−6.1
	Mammalian target of rapamycin	mTOR	1aue		−8.5

**Fig. 2:**
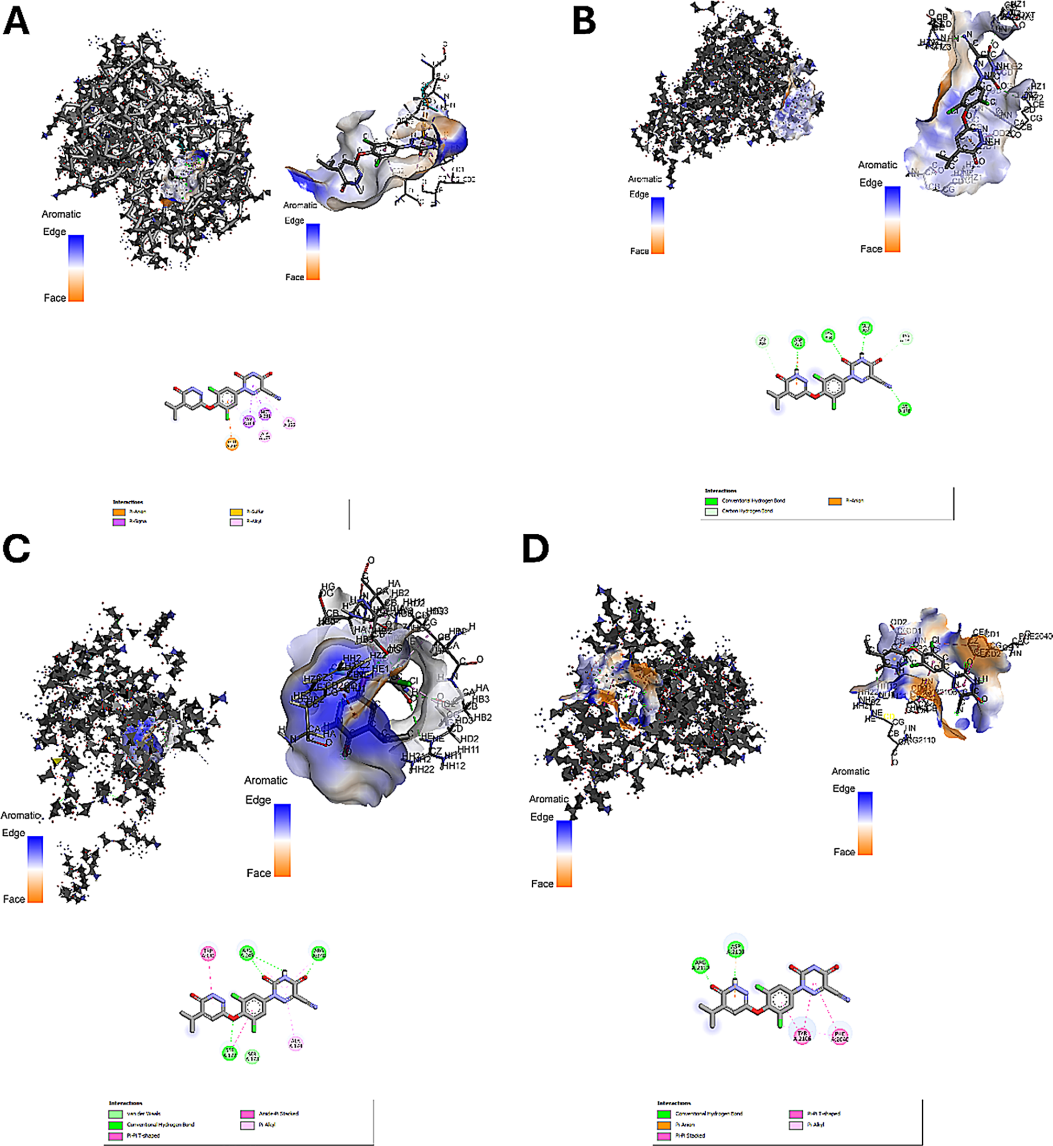
Molecular docking results of resmetirom with (**A**) AKT1 (PBD ID: 6ccy), (**B**) DRP1 (PBD ID: 5ons), (**C**) FOXO1 (PBD ID: 6qvw), and (**D**) mTOR (PBD ID: 1aue). AKT Serine/Threonine kinase 1 (AKT1), Dynamin-related protein 1 (DRP1), Forkhead box protein O1 (FOXO1), and Mammalian target of rapamycin (mTOR)

According to the molecular docking research, the binding affinities for the interactions between RES and AKT1, DRP1, FOXO1, and mTOR were −8.6, −6.2, −6.5, and −7.2 kcal/mol, respectively (Table 3; Fig. 2).

**Table 3:** Molecular docking procedure involving the resmetirom molecule and key targets

Compound name	Targets	Gene name	PDB number		Binding AFFINITIES (kcal/mol)
resmetirom (RES)	AKT Serine/Threonine Kinase 1	AKT1	6ccy		−8.6
	Dynamin-related protein 1	DRP1	5ons		−6.2
	Forkhead box protein O1	FOXO1	4eo7		−6.5
	mammalian target of rapamycin	mTOR	1aue		−7.2

### Renal function parameters evaluations

**Fig. 3:**
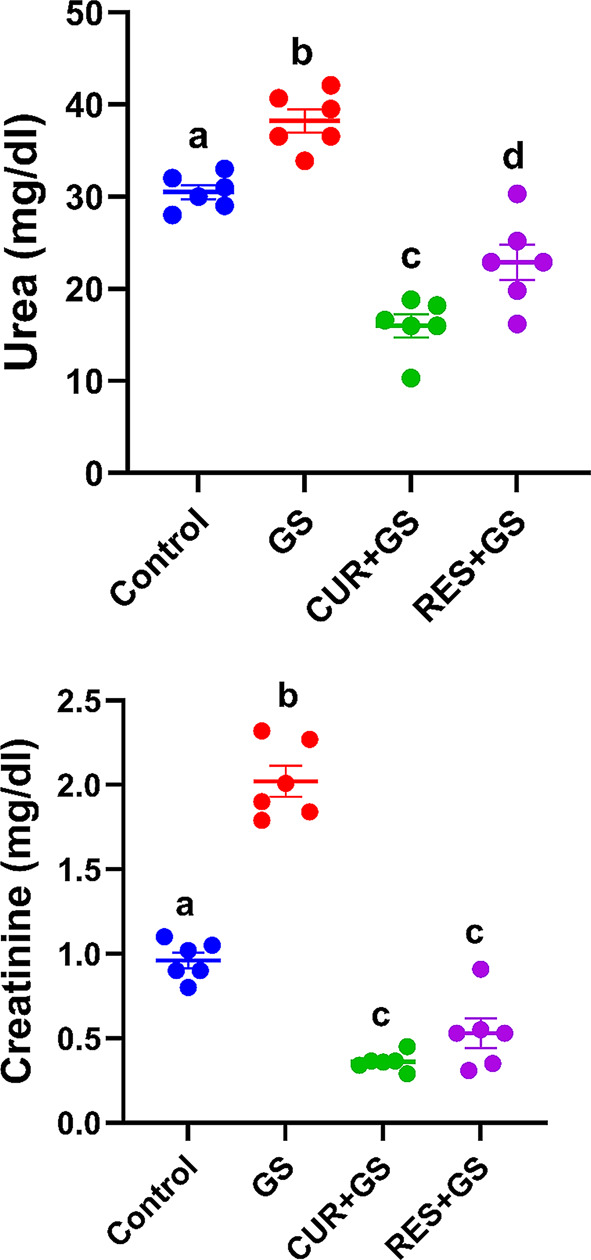
The effects of curcumin (CUR) and resmetirom (RES) treatment on serum levels of urea and creatinine in rats during gentamicin (GS)-induced nephrotoxicity. Data expressed as mean ± SEM (*n* = 6/group). Different letters indicate significant differences among treatments (*p* < 0.05) (one-way ANOVA, followed by Tukey post hoc test)

When compared to the control group, the group that received GS had significantly higher serum concentrations of urea and creatinine (*p* < 0.0001, *p < 0.01,* respectively). The levels were significantly lower (*p* < 0.0001) in rats treated with CUR and RES compared to rats treated with GS, with the rats treated with CUR showing the best improvement (Fig. 3).

### Oxidative stress markers evaluations

**Fig. 4:**
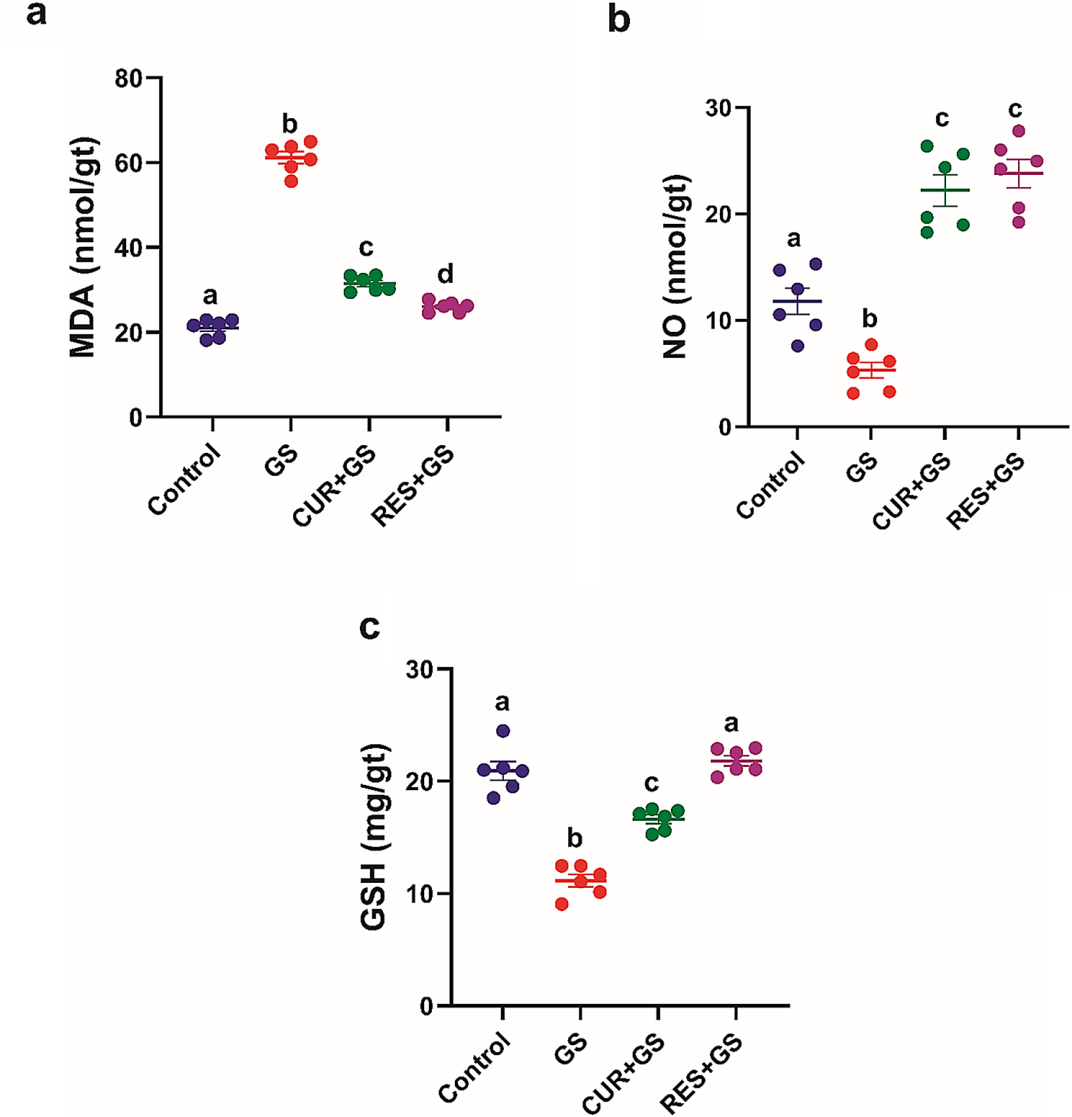
The effects of curcumin (CUR) and resmetirom (RES) treatment on renal tissue levels of malondialdehyde (MDA), nitric oxide (NO), and reduced glutathione (GSH) in rats during gentamicin (GS)-induced nephrotoxicity. Data expressed as mean ± SEM (*n* = 6/group). Different letters indicate significant differences among treatments (*p* < 0.05) (one-way ANOVA, followed by Tukey post hoc test)

In comparison to the control group, the GS group had markedly elevated tissue concentrations of MDA (*p* < 0.0001). The levels were markedly reduced (*p* < 0.0001) in rats administered CUR and RES in comparison to those treated with GS, with the CUR group exhibiting the most substantial enhancement (Fig. 4a).

In comparison to the control group, the GS group exhibited significantly decreased tissue concentrations of NO and GSH (*p* < 0.01, *p* < 0.0001, respectively). The levels were markedly elevated (*p* < 0.0001) in rats administered CUR and RES relative to those treated with GS, with the RES group exhibiting the most substantial enhancement (Fig. 4b, c).

### Assessments of KIM-1 and NGAL renal tissue levels

**Fig. 5:**
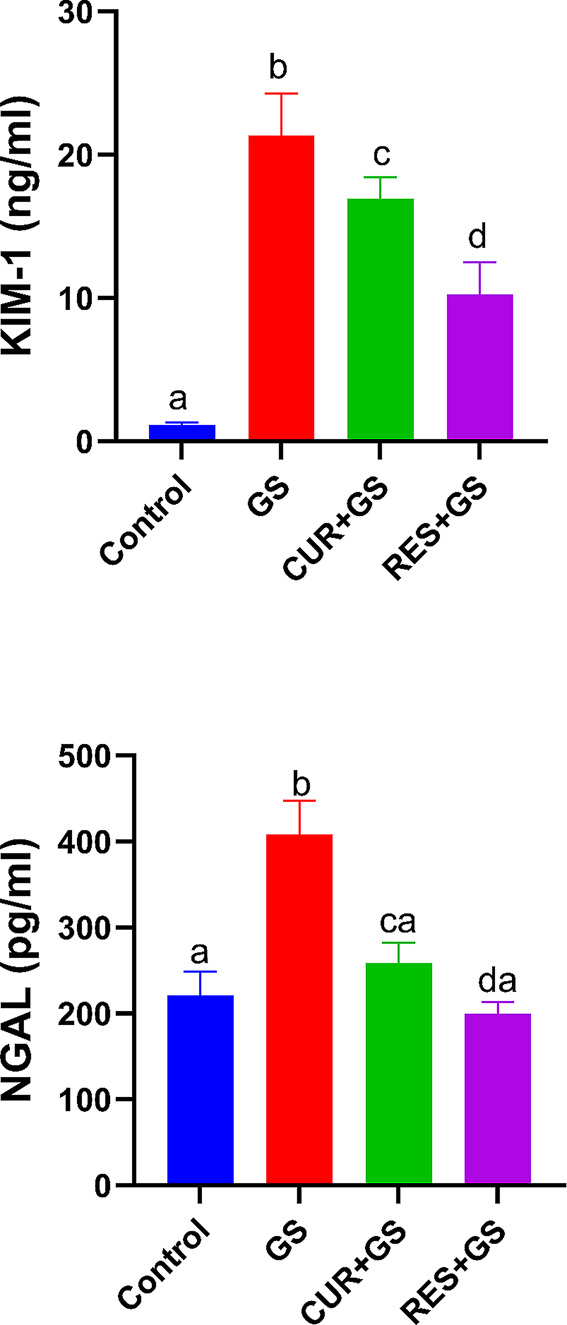
The effects of curcumin (CUR) and resmetirom (RES) treatment on renal tissue levels of kidney injury molecule-1 (KIM-1) and neutrophil gelatinase-associated lipocalin (NGAL) in rats during gentamicin (GS)-induced nephrotoxicity. Data expressed as mean ± SEM (*n* = 6/group). Different letters indicate significant differences among treatments (*p* < 0.05) (one-way ANOVA, followed by Tukey post hoc test)

Tissue levels of KIM-1 and NGAL were markedly elevated in the GS-treated rat group relative to the control group (*p* < 0.0001). Relative to the GS-induced renal injury group, CUR and RES markedly reduced tissue levels of KIM-1 and NGAL (*p* < 0.0001), with the RES group demonstrating the most pronounced enhancement (Fig. 5).

### Quantitative PCR assessment for DRP1 and AKT1

**Fig. 6:**
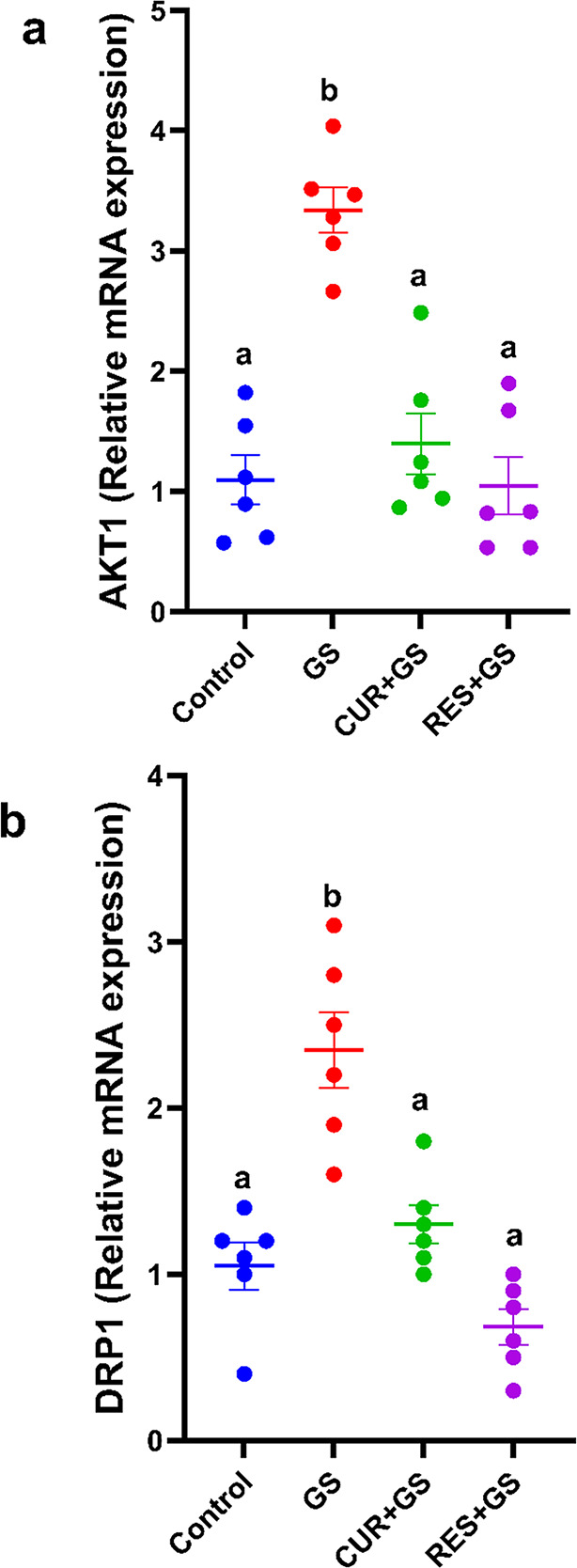
Relative gene expression of AKT Serine/Threonine kinase 1 (AKT1), Dynamin-related protein 1 (DRP1) in all groups studied. β-actin was employed to standardize expression data. *n* = 6 for each group. Data is presented as mean ± SEM. Different letters indicate significant differences among treatments (*p* < 0.05) (one-way ANOVA, followed by Tukey post hoc test). Gentamicin (GS), curcumin (CUR) and Resmetirom (RES)

Cell growth and survival protein AKT1 expression was considerably elevated (*p* < 0.0001) in GS-treated rats compared to the control, as illustrated in Fig. 6a. Conversely, CUR and RES markedly reduced (*p* < 0.0001) AKT1 expression relative to rats in the GS-treated group.

As illustrated in Fig. 6b, the expression of the essential regulator of mitochondrial fission, DRP1, was considerably (*p* < 0.0001) elevated in GS-treated rats compared to the control group. Conversely, CUR and RES markedly reduced (*p* < 0.0001) DRP1 expression relative to the rats in the GS-treated group.

### Histopathological evaluations

**Fig. 7:**
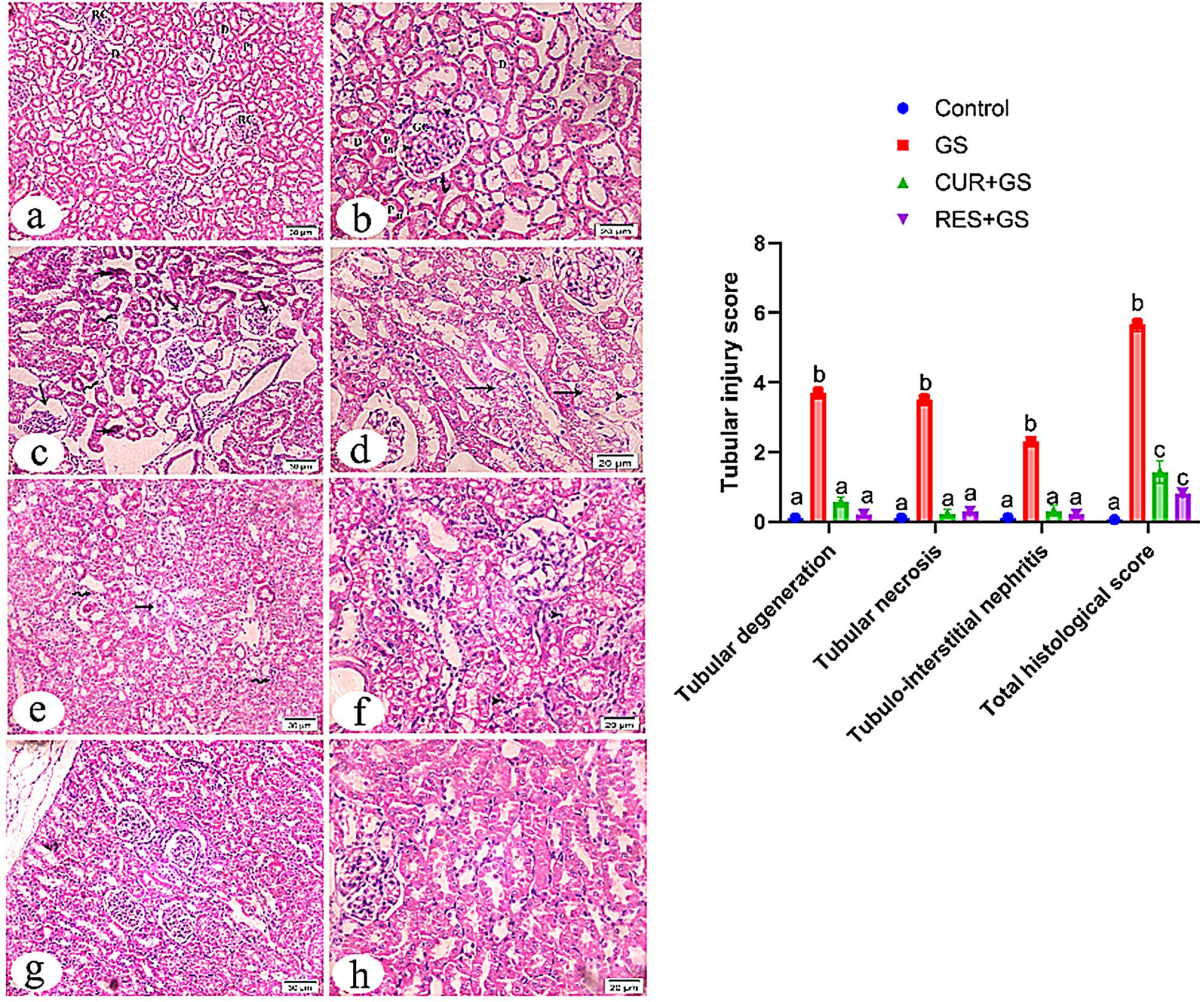
Light photomicrographs of rat’s kidney tissue sections of all study groups stained with hematoxylin and eosin (H&E) and semi-quantitative measurement of tubular injury score. **a**. low magnification showing the histological structure of kidney cortex in control group is occupied by renal corpuscles (RC), proximal tubules (P) and distal tubules (D). **b**. in higher magnification of RC revealing a central tuft of looped blood capillaries (GC) surrounded by Bowman’s capsule. This capsule is formed of two; outer parietal layer (curved arrow) of and the inner, visceral layer that is comprised of podocytes (arrow heads). The renal tubules showing the normal histology of proximal tubules (P) with vesicular rounded nuclei (**n**), deep acidophila, narrow lumen. In addition to the distal tubules (D) that are larger in size and wider in lumen than the proximal tubules. **c**. the kidney cortex in gentamicin (GS)-induced nephrotoxicity showing number of disorganized renal corpuscles (arrows). Bowman’s spaces are irregular and enlarged with shrinkage glomerular capillaries and intraglomerular mesangial hypercellularity. In addition to many destructed renal tubules (short arrows) with obliterated lumen and dense nuclei surrounded by diffuse foci of cellular infiltration (wavy arrows) are noticed. **d**. in higher magnification showing destructed proximal and distal tubular damages (arrows) such as cytoplasmic vacuolization and loss of brush border with dense nuclei (arrow heads) of some cells are noticed. **e**. the organization of renal cortex is apparently restored to normal structure in GS+ curcumin except for few distorted renal corpuscles (arrow) adjacent to some cellular infiltration (wavy arrows). **f**. in higher magnification of renal tubules showing many restored tubules, few of them with vacuolated cytoplasm and dense nuclei (arrow heads). **g**. & **h**. in GS+ Resmetirom, there is more or less no apparent histopathological changes in the structure of kidney cortex which are equal near to the control rats

The histological data for all investigated groups indicate the lesion tubular injury scores, as presented in Fig. 7. The H&E-stained tissue slices from the control group exhibited the characteristic histological architecture of the kidney cortex, comprising renal corpuscles, proximal tubules, and distal tubules (Fig. 7a). The renal corpuscle consisted of a central tuft of looped blood capillaries located in the center of the renal corpuscle surrounded by Bowman’s capsule. The blood capillaries are formed of a single layer of endothelial cells resting on a basement membrane. The capsule is formed of the outer parietal layer of simple flattened (squamous) epithelium and the inner visceral epithelium that is comprised of podocytes. These cells appear with rounded vesicular nuclei related to the glomerular capillaries. The renal tubules revealed normal histology of proximal tubules with vesicular rounded nuclei, deep acidophilic cytoplasm, and a narrow lumen with a brush border. In addition, the distal tubules were larger in size and wider in lumen than the proximal tubules, with more rounded nuclei, lighter acidophilic cytoplasm, and fewer brush borders (Fig. 7b).

In tissue sections from the GS-treated group, a number of the renal corpuscles lost their normal size and shape. Bowman’s spaces are irregular and enlarged with shrinkage and disorganization of the glomerular capillaries’ structure in the corpuscles with intra-glomerular mesangial hypercellularity (Fig. 7c). In addition, many destructed renal tubules with obliterated lumens and dense nuclei surrounded by diffuse foci of leukocytic infiltration were noticed. Increased edematous spacing was observed in the interstitial areas around degenerated renal corpuscles and tubules. Also, the destructed proximal tubular damage, such as cytoplasmic vacuolization and loss of brush border with dense nuclei of some cells, was noticed. In addition to many irregularities, dilated distal tubules with marked vacuolated cytoplasm were revealed (Fig. 7d).

The organization of the renal cortex is more or less restored in the CUR-GS treated group except for a few distorted renal corpuscles. The renal tubules exhibited numerous restored structures, with some displaying vacuolated cytoplasm and dense nuclei. Brush border was apparently restored to normal shape in most of the tubules. Residual cellular infiltration was seen (Fig. 7e, f).

The RES-GS treated group showed no apparent histopathological changes in the structure of the kidney cortex, which was nearly equal to that of the control rats. Minimal cellular infiltration was noticed (Fig. 7g, h).

### Immunohistochemical and morphometric evaluations

**Fig. 8:**
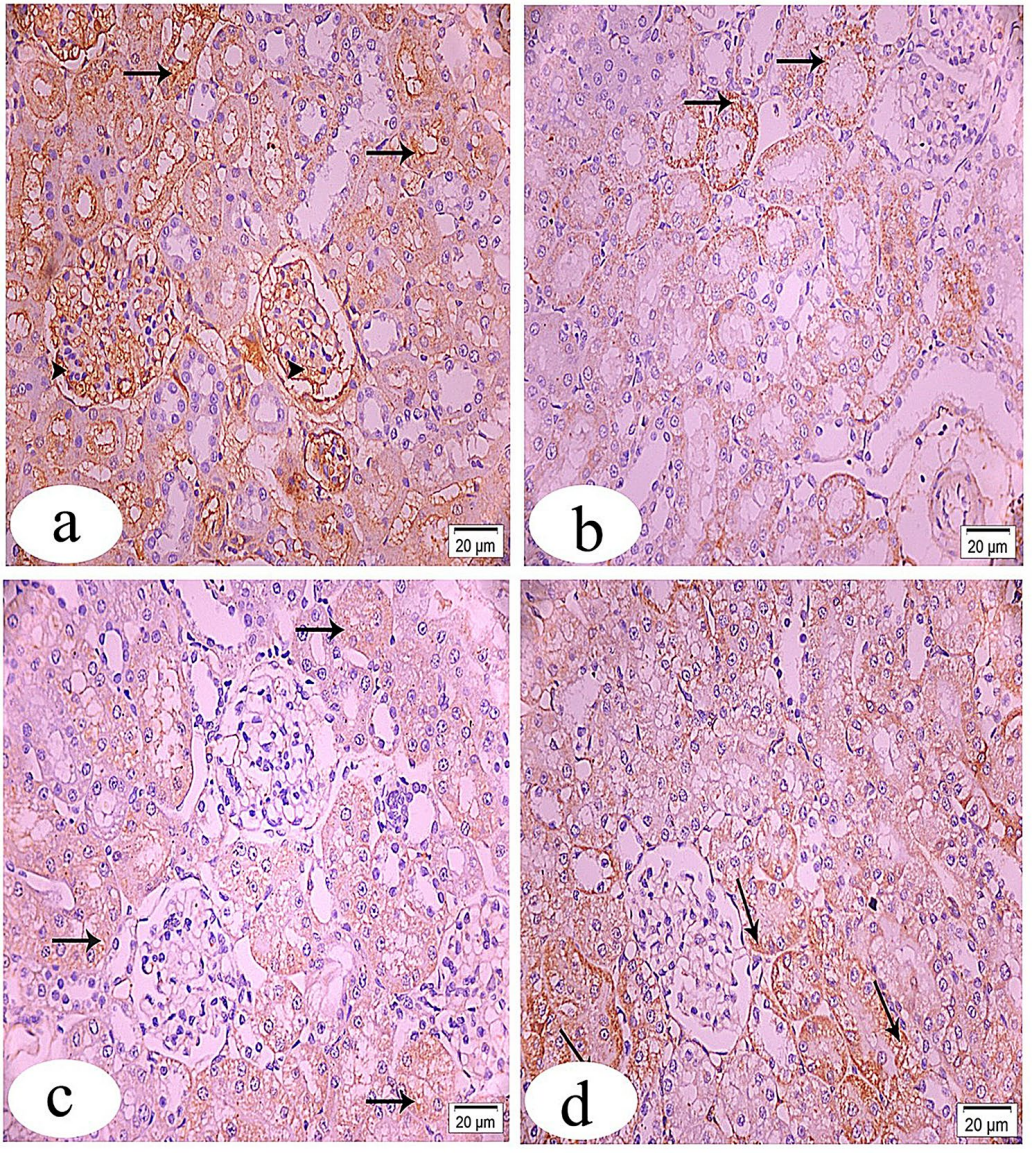
Light photomicrographs of rat’s kidney tissue sections stained with FOXO1 immunostaining at x400 magnification. **a**. control group showing strong positive immunostaining reaction within the cytoplasm of many glomerular (arrow heads) and tubular (arrows) cells. **b**. gentamicin (GS)-induced nephrotoxicity group showing faint positive immunostaining reactivity of some tubular cells (arrows) in addition to increasing immunostaining area within the cytoplasm of many glomerular cells (arrow heads). **c**. GS+ curcumin group exhibiting moderate increase in positive immuno-expression in both glomerular (arrow heads) and tubular cells (arrows) compared to group II. **d**. GS+ Resmetirom group revealing marked increase in the positive cytoplasmic immunostaining especially of tubular cells (arrows)

Mammalian target of rapamycin immunostaining sections of the control group showed strong positive cytoplasmic immune expression of many glomerular and tubular cells (Fig. 8a). The immunostaining reactivity was decreased in almost all of the tubules, with a few positive staining cells in the GS-treated group (Fig. 8b). In the CUR-GS & RES-GS groups, respectively, there was an increase in positive immunostaining area within the cytoplasm of many tubular cells compared to the GS-treated group (Fig. 8c, d).

**Fig. 9:**
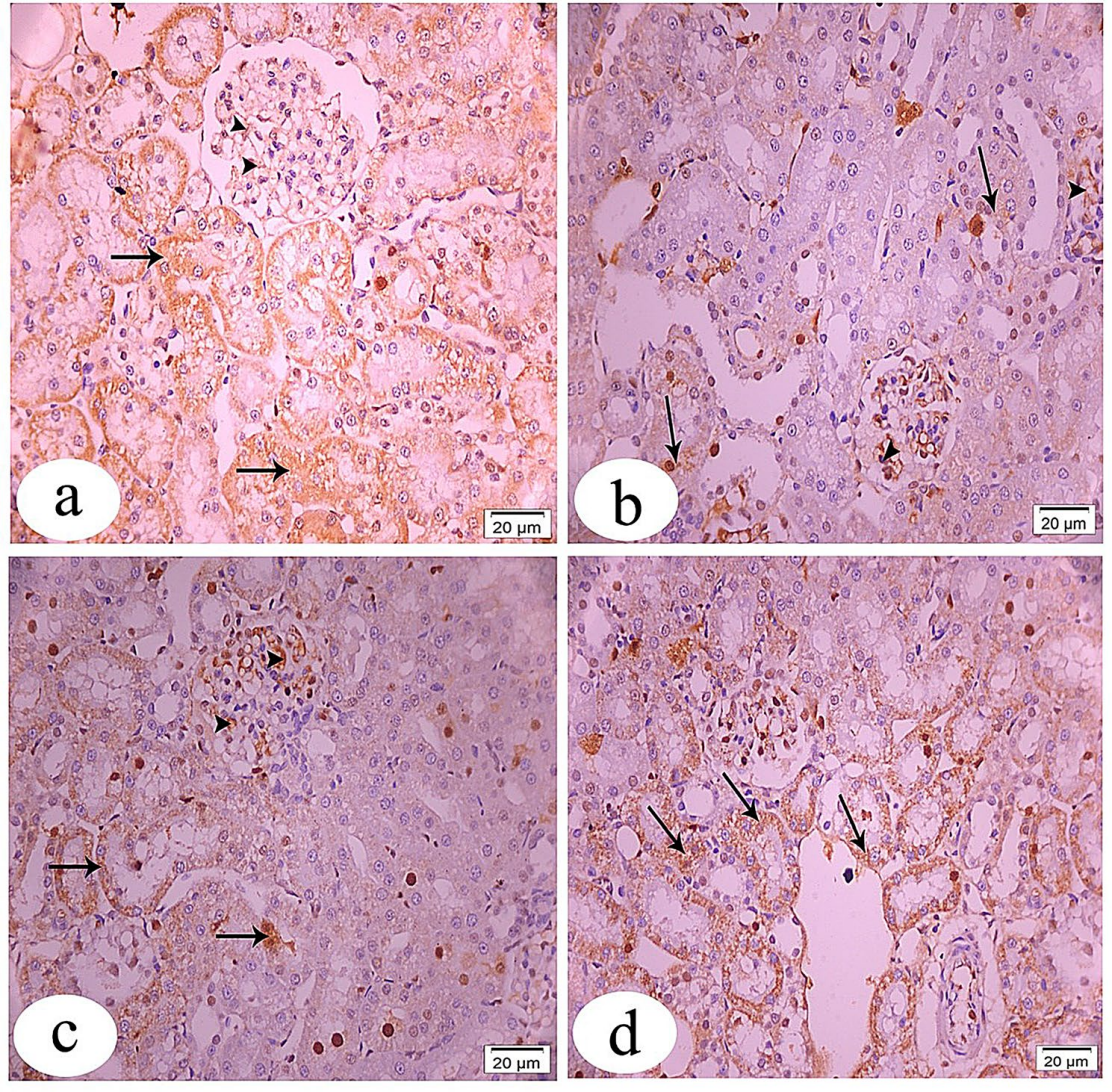
Light photomicrographs of rat’s kidney tissue sections stained with mTOR immunostaining at x400 magnification. **a**. control group showing strong positive cytoplasmic immune expression of many glomerular (arrow heads) and tubular (arrows) cells. **b**. the immunostaining reactivity is decreased in almost of the tubules with few positive staining cells (arrows) in gentamicin (GS)-induced nephrotoxicity group. **c** & **d**. GS+ curcumin group & GS+ Resmetirom group respectively, revealing increase in positive immunostaining area within the cytoplasm of many tubular cells (arrows) compared to group II

Forkhead box protein O1 immunostaining of a section of the control group showed a strong positive immunostaining reaction within the cytoplasm of many glomerular and tubular cells (Fig. 9a). In the GS-treated group, the section showed faint positive immunostaining reactivity of some tubular cells, in addition to increased immunostaining area within the cytoplasm of many glomerular cells (Fig. 9b). The CUR-GS group exhibited a moderate increase in positive immunoexpression in both glomerular and tubular cells compared to the GS-treated group (Fig. 9c). In the RES-GS group, the section revealed a marked increase in the positive cytoplasmic immunostaining, especially of tubular cells (Fig. 9d).

**Fig. 10:**
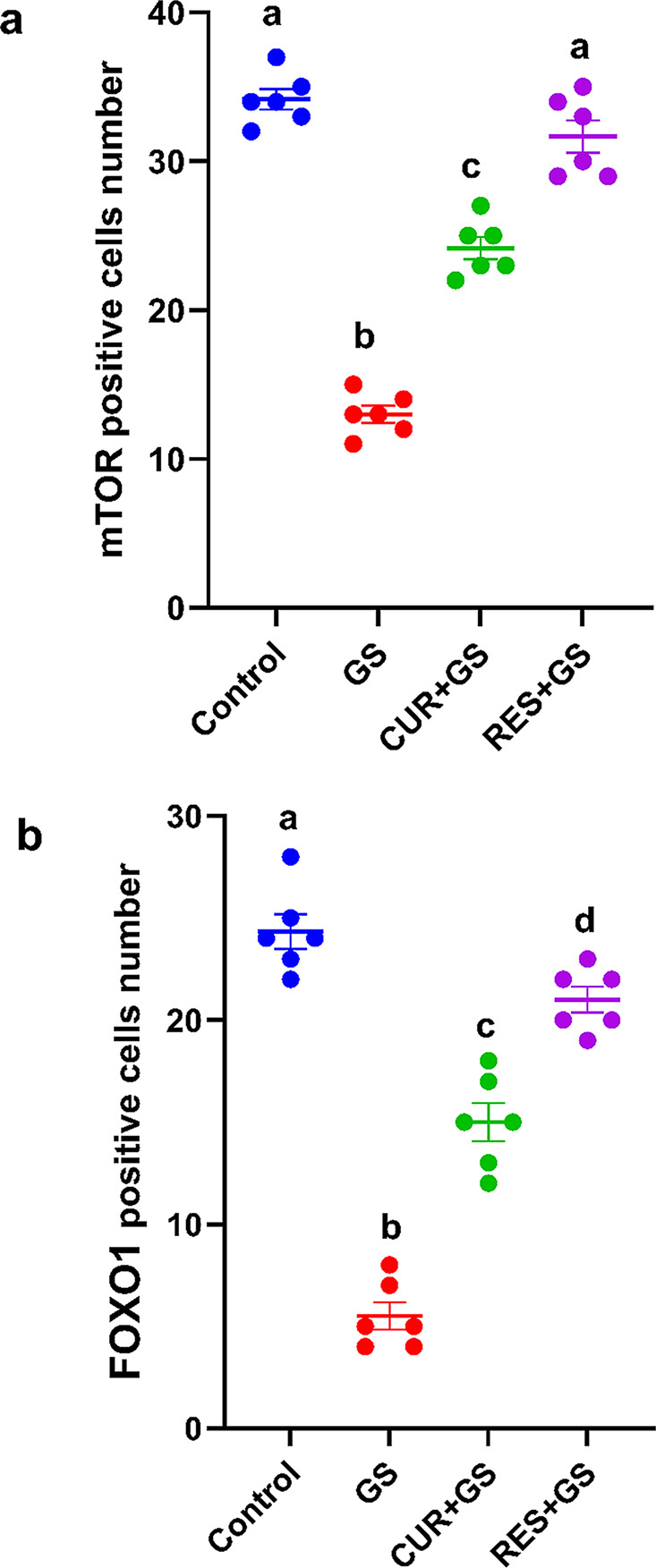
Quantitative measurement of positive immunohistochemistry staining. **a**. Graph depicting the quantification of mTOR immunopositive cells across all groups, **b**. Graph illustrating the quantification of FOXO1 immunopositive cells across all groups. Different letters indicate significant differences among treatments (*p* < 0.05) (one-way ANOVA, followed by Tukey post hoc test). Gentamicin (GS), curcumin (CUR) and Resmetirom (RES)

Protein immune expression of both mTOR and FOXO1 was dramatically reduced (*p* < 0.0001) in the GS-treated group compared to the control group. Treatment with either CUR or RES significantly (*p* < 0.0001) elevated their levels relative to the GS-treated group, with a more pronounced enhancement observed in the RES-GS group (Fig. 10).

### Electron microscopic evaluations

#### Semithin evaluations

**Fig. 11:**
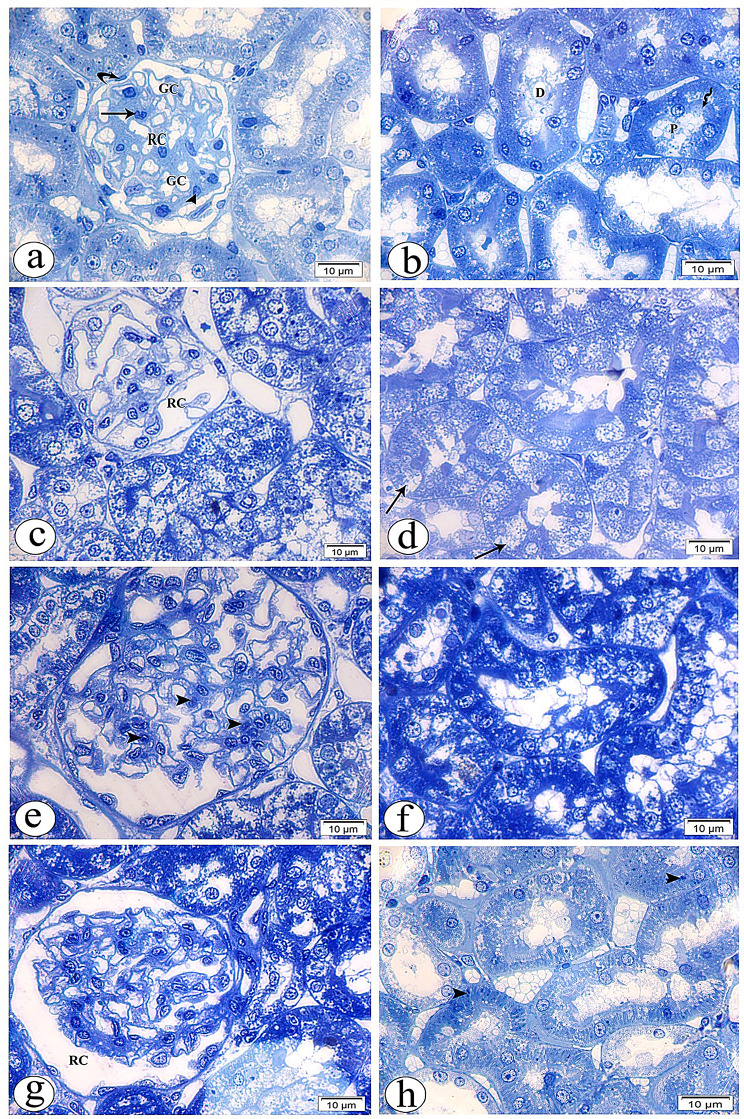
Light photomicrographs of rat’s kidney tissue semithin sections of all study groups stained with toluidine blue (x1000). Control group showing the classical organization of renal corpuscle (RC) consist of central regular spacing of glomerular capillaries (GC) surrounded by podocytes (arrow head). Mesangial cells (arrow) and parietal layer (curved arrow) of Bowman’s capsule are seen (**a**). The normal structure of renal proximal (P) and distal (D) tubules are shown, brush borders are evident (wavy arrow) (**b**). Gentamicin (GS)-induced nephrotoxicity group showing degenerated renal corpuscle (RC) with shrinkage of capsular contents that surrounded by irregular capsular space (**c**). In addition to, many tubular cells (arrows) with pale vacuolated cytoplasm and disorganized brush borders are noticed (**d**). GS+ curcumin group reveals normal structure of renal corpuscle with some increase in the number of mesangial cells nuclei (arrow heads) in between the capillary spacing (**e**). some tubular changes are still seen in the cells of renal tubules (**f**). GS+ Resmetirom group showing more or less normal configuration and cytoarchitecture of renal corpuscle (RC) and tubules with many dense granules (arrow heads) in the cytoplasm of some tubular cells and restored brush borders are seen (**g** & **h**)

The control group showed the classical organization of the renal corpuscle (RC), consisting of central spacing of glomerular capillaries lined by simple squamous endothelial cells surrounded by podocytes that contain large rounded vesicular nuclei with pale basophilic cytoplasm. Small, deeply stained nuclei of mesangial cells invested in between the capillaries are seen in sites not covered by podocytes. Also, the regular capsular spacing and the simple flattened epithelium (parietal layer) of Bowman’s capsule were revealed. The normal structure of renal proximal convoluted tubules was rounded in shape and contained large rounded vesicular basal nuclei of high pyramidal cells, leaving a narrow lumen; a brush border was evident. In contrast, the distal one was tubular in shape with more rounded nuclei and low pyramidal cells, leaving a wide lumen. Both of them rest on the basement membrane (Fig. 11a, b).

The gentamicin sulfate-treated group showed degenerated RC with shrinkage of capsular contents that were surrounded by irregular wide capsular space. The nuclei of podocytes and mesangial cells showed abnormal staining properties with a notable increase in the number of mesangial cells relative to podocytes. In addition, many cells of both proximal and distal tubules with pale vacuolated cytoplasm were noticed. Moreover, lost and disorganized brush border was revealed (Fig. 11c, d).

The CUR-treated group revealed a normal structure of the renal corpuscle with some increase in the number of mesangial cell nuclei in between the capillary spacing. Some tubular changes are still seen in the cells of renal tubules with apparent normal structure; the brush border was apparently normal in some tubules (Fig. 11e, f).

The RES-treated group showed more or less normal configuration and cytoarchitecture of RC and tubules with many scattered dense granules in the cytoplasm of some tubular cells seen. Brush border was apparently normal (Fig. 11g, h).

#### Imaging using an electron microscope

##### Renal corpuscle

**Fig. 12:**
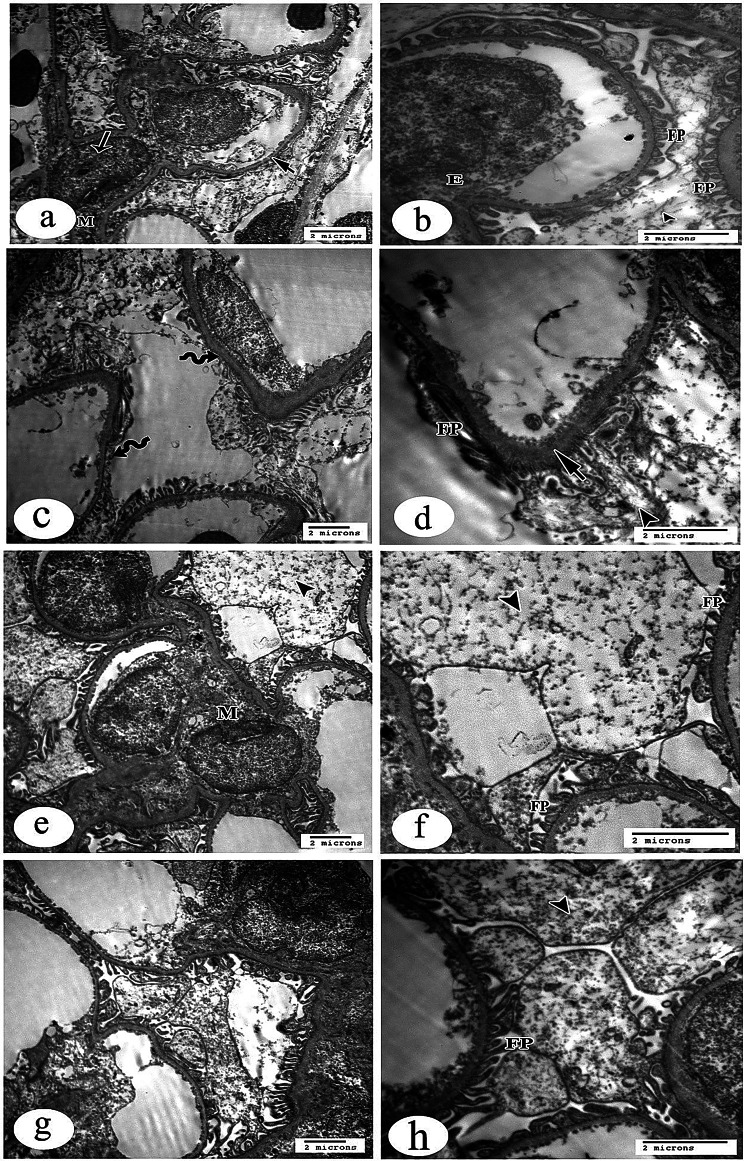
Electron micrographs of proximal convoluted tubules (PCT) from rat kidney tissue sections (X 4800). **a**. control group showing the luminal surface of the PCT has well-developed microvilli forming brush border (arrow). Endocytic vesicles and lysosomes are observed in the apical cytoplasm beneath the microvilli. Numerous mitochondria (M) are also observed distributed all over the cytoplasm. The nuclei (N) of these cells are relatively large, mostly euchromatic. Notice normally evident basement membrane (BM) **b**. gentamicin (GS)-induced nephrotoxicity group showing apparent changes in their ultrastructural configuration. The microvilli constituting the brush border of the lining cells are disorganized and partially degenerated (arrow). Aggregations of many vacuoles near the basal part of the microvilli are also observed within the allover rarified cytoplasm. In addition to that, many mitochondria are swollen with destructive matrices (arrow heads) seen. Notice thin basement membrane (BM) **c**. & **d**. GS+ curcumin group & GS+ Resmetirom group respectively, showing quite normal appearance of PCT except few destructed mitochondria (arrow heads) are noticed in the cytoplasm. Notice apparent normally evident basement membrane (BM)

Sections of the control group of renal glomeruli showed spaces of capillary tufts surrounded by glomerular basement membrane (GBM); these tufts were separated by normal mesangial cells and their matrix (Fig. 12a). Renal glomerulus revealed a renal capillary with its nucleus of endothelial cells and intact foot processes of podocyte cells that rest on the urinary side of GBM (Fig. 12b)**.**

Gentamicin sulfate-treated group sections displayed spaced glomerular capillary loops floating in the urinary space with features of podocyte injury. The renal capillary with thickening of GBM and abnormal irregular fused foot processes was shown, thus obliterating the infiltration slits of the surrounded damaged podocyte and giving them a disorganized arrangement (Fig. 12c, d)**.**

Curcumin-treated group showed more or less normal configuration of renal glomerulus that consisted of spaces of renal capillaries surrounded by podocytes and its processes that were separated by mesangial cells and its dark matrix. Also, part of a normal podocyte and its intact foot processes, which rest on GBM was displayed. The GBM was of apparent normal thickness (Fig. 12e, f).

Resmetirom-treated group sections showed quite apparent normal renal glomeruli and podocytes with their intact foot processes and apparent normal thickness of the GBM (Fig. 12g, h).

##### Proximal convoluted tubule (PCT)

**Fig. 13:**
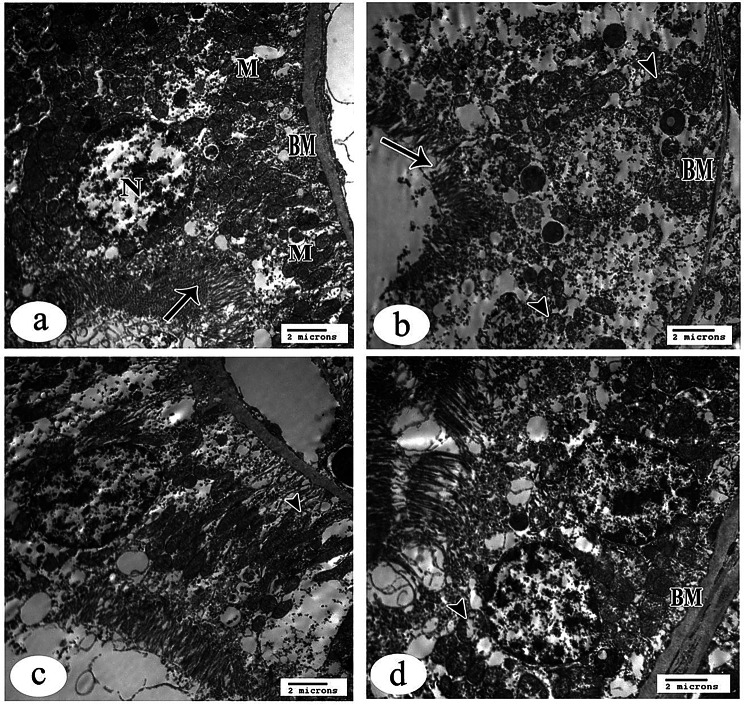
Electron micrographs of distal convoluted tubules (DCT) from rat kidney tissue sections (X 4800). **a**. control group showing DCT is lined by cuboidal epithelial cells. The cells do not have a brush border (arrow) and instead, they show short scanty microvilli at the apical membrane. The small and round mitochondria (M) are distributed within the cytoplasm especially at the basal portion. The nuclei (N) are large, and their heterochromatin appears adherent to the inner surface of the nuclear envelope. **b**. gentamicin (GS)-induced nephrotoxicity group showing the cytoplasm appears to be rarified and has many disintegrated mitochondria. These mitochondria lost their matrices (arrow heads) so that they did not show any demarcation of their detailed fine structures. The nuclei (N) of affected cells revealed irregular outlines with some dilated pores of its nuclear envelope. **c**. GS+ curcumin group showing loss of some mitochondria within quite empty rarified cytoplasm. **d**. GS+ Resmetirom group showing the cells of DCT are apparently near to control without evident pathological findings

The brush border is formed by well-developed microvilli on the luminal surface of the PCT, as shown in sections of the control group. The apical cytoplasm, just below the microvilli, contains lysosomes and endocytic vesicles. There are many mitochondria scattered throughout the cytoplasm as well. This cell type often has big, mainly euchromatic nuclei. No damage was found to the basement membrane (Fig. 13a, b).

There were noticeable alterations in the ultrastructural configuration of the group treated with GS. The brush border of the lining cells, which is made up of microvilli, was largely deteriorated and disordered. Within the allover rarefied cytoplasm, there are also vacuole aggregates close to the base of the microvilli. Not only that, but a large number of mitochondria have enlarged and damaged matrices. Thin basement membrane was seen (Fig. 13c, d).

In the CUR-GS and RES-GS treated groups, the appearance of PCT was quite normal except for a few destructive mitochondria noticed in the cytoplasm. The restored apparent normal basement membrane was noticed (Fig. 13e, f, g, and h).

##### Distal convoluted tubule (DCT)

**Fig. 14:**
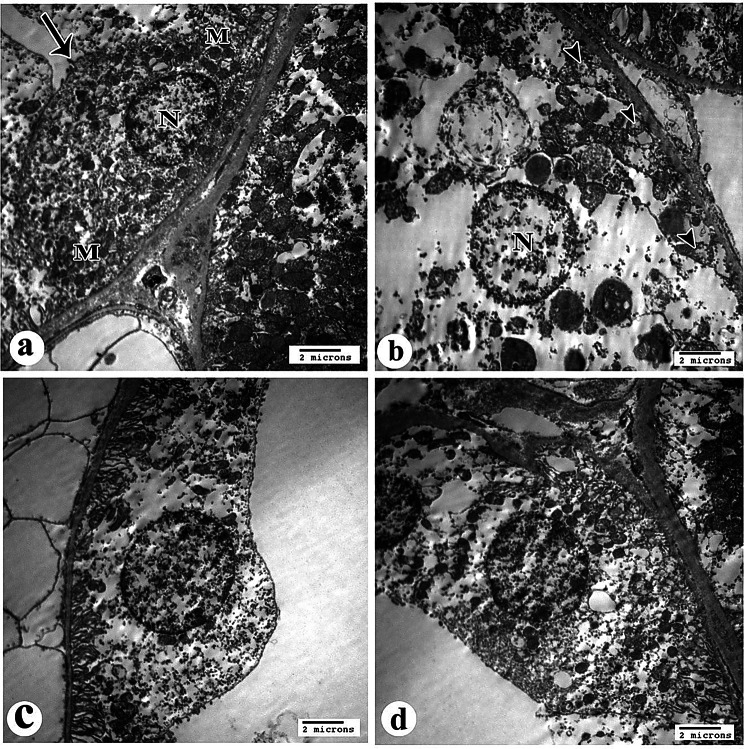
Electron micrographs of capillary loops of a glomerulus from rat kidney tissue sections. **a**. control group of renal glomerulus showing spaces of capillary tufts surrounds by glomerular basement membrane (GBM) (short arrow), and normal mesangial cells (arrow) and its matrix (M) (X 4800). **b**. control group renal glomerulus showing higher magnification of renal capillary with its nucleus of endothelial cell(E) and intact foot processes (FP) of podocyte (arrowhead) that rest on the urinary side of (GBM) (x10000). **c**. Gentamicin (GS)-induced nephrotoxicity group showing spaced glomerular capillary loops (wavy arrows) that floating in the urinary space with features of podocytes injury (X 4800). **d**. GS-induced nephrotoxicity group showing higher magnification of renal capillary with thickening of GBM (short arrow), and abnormal irregular fused foot processes (FP), thus obliterating the infiltration slits of surrounded damaged podocyte (arrowhead) (x10000). **e**. GS+ curcumin group showing more or less normal configuration of renal glomerulus that consist of spaces of renal capillaries surround by podocytes (arrowhead) and its processes that separated by mesangial cells and its dark matrix (M) (X 4800). **f**. GS+ curcumin group showing higher magnification of part of normal podocyte cell (arrowhead) and its intact foot processes (FP), which rest on GBM with apparent normal thickness (x10000). **g**. GS+ Resmetirom showing quite normal renal glomerulus with apparently normal thickness of the GBM (X 4800). **h**. GS+ Resmetirom showing normal podocyte (arrowhead) and its intact foot processes (FP) in higher magnification (x10000)

The DCT lining was observed in the control group to be cuboidal epithelial cells. Instead of a brush border, the cells displayed short and sparse microvilli at their apical membrane. Throughout the cytoplasm, particularly at the base, were the little, spherical mitochondria. Their heterochromatin seemed to be stuck to the inside of the nuclear envelope, and the nuclei were huge (Fig. 14a, b).

The cytoplasm seemed rarified and had numerous disintegrating mitochondria in the group treated with gentamicin sulfate. These mitochondria were unable to display their intricate fine architecture because they had lost their matrices. Injured cells’ nuclei showed distorted shapes and enlarged nuclear envelope holes (Fig. 14c, d). In the CUR-GS treated group, loss of some mitochondria within quite empty rarified cytoplasm (Fig. 14e, f). In the RES-GS treated group, the cells of DCT are apparently near to control without evident pathological findings (Fig. 14g, h).

## Discussion

Gentamicin, a commonly given aminoglycoside antibiotic, demonstrates significant efficacy against Gram-negative bacterial infections. However, its therapeutic application is constrained by pronounced nephrotoxic and ototoxic effects, which may need cessation in extreme instances [39]. GS-induced nephrotoxicity primarily affects the renal proximal tubules, leading to structural damage, functional impairment, and oxidative stress. High GS concentrations not only trigger apoptotic pathways but also necrotic cell death through ATP depletion and lysosomal proteolysis, exacerbating kidney damage [40]. Clinically, this damage presents as acute kidney injury, which may advance to chronic kidney disease with extended or recurrent exposure [6]. Pathogenesis involves reactive oxygen species generation, lipid peroxidation, mitochondrial dysfunction, and activation of inflammatory pathways [41, 42].

Xenobiotics, heavy metals, chemotherapeutic drugs, and antibiotics can cause nephrotoxicity, which is a major problem in clinical practice because of the many pathways involved, such as oxidative stress, inflammation, necroptosis, apoptosis, and transporter-mediated accumulation. An active ingredient of curcuma longa, CUR reduces nephrotoxic kidney damage through its antioxidant, anti-inflammatory, anti-fibrotic, and anti-apoptotic capabilities [43]. Synthetic curcumin analogs have recently shown promise as nephroprotective drugs due to their increased efficacy compared to curcumin, frequently at lower doses. Nevertheless, additional study is necessary to confirm their safety and effectiveness in humans, as there have been no large-scale clinical trials [44].

A new medicine RES was approved by the FDA in March 2024 to treat steatohepatitis in people who do not have cirrhotic metabolic dysfunction. RES’s anti-inflammatory, antifibrotic, and antioxidant properties make it a potential candidate for lowering GS-induced kidney damage [21]. Kidney disease and nonalcoholic fatty liver disease share many pathophysiological features and risk factors, and both organs have significant numbers of thyroid hormone beta receptors. This suggests that thyroid hormone beta receptor agonists may be useful in the treatment of chronic renal illness. Recent study has demonstrated that metabolic-associated fatty liver disease not only impacts liver prognosis but also increases the likelihood of developing other chronic diseases, such as cardiovascular disease, chronic kidney disease, and non-hepatic malignancies [20].

The present study provides a comprehensive evaluation of GS-induced nephrotoxicity through the integration of biochemical, histopathological, immunohistochemical, and ultrastructural analyses. GS was administered at a well-established nephrotoxic drug known to induce acute kidney injury, enabling detailed characterization of renal damage at both cellular and molecular levels [45]. The doses of CUR and RES were chosen to maximize therapeutic efficacy while avoiding potential adverse effects, providing a rational foundation for their use as renoprotective agents against GS-induced nephrotoxicity.

Serum urea and creatinine were selected as major renal biomarkers at the biochemical level because of their recognized clinical significance and sensitivity in identifying functional kidney impairment. Both values are well-known measures of glomerular filtration rate and are commonly used in clinical and experimental tests of kidney function. High blood urea and creatinine levels show that the kidneys are not clearing waste properly. This is a sign of nephrotoxicity, especially in drug-induced kidney injury models like GS toxicity. The addition of these biomarkers provides a swift and non-invasive method to evaluate the extent of renal impairment and the effectiveness of preventative measures, thereby augmenting the translational significance of the results [46].

The administration of GS alone in this investigation led to a considerable increase in serum urea and creatinine levels compared to the control group, validating the induction of nephrotoxicity. These results corroborate prior research indicating that GS primarily induces acute kidney injury through pathways associated with oxidative stress, inflammation, and tubular necrosis, especially within the proximal convoluted tubules [47]. Co-administration of CUR considerably mitigated these changes, as seen by marked decreases in serum urea and creatinine concentrations. CUR is known for its strong antioxidant, anti-inflammatory, and anti-apoptotic properties, which may be what makes it good for the kidneys [48]. Its ability to scavenge reactive oxygen species, modulate nuclear factor-kappa B signaling, and suppress pro-inflammatory mediators provides a plausible mechanistic basis for the observed improvements in renal function parameters [49]. A stronger protective effect was shown with RES therapy compared to CUR. Greater decreases in serum urea and creatinine, as shown by boxplots with lower median values and smaller interquartile ranges, provided clear evidence of this.

Oxidative stress is an important factor in the worsening of nephrotoxicity caused by GS [50]. The increase in MDA levels induced by GS in this investigation was substantially mitigated by both CUR and RES. Moreover, the reduced NO levels induced by GS were further exacerbated by both CUR and RES. Consistent with previous researchers, who demonstrated the nephrotoxic effects of GS via an oxidative stress mechanism by increasing MDA levels and reducing superoxide dismutase and glutathione levels [42, 50]. Prior studies validated the potent antioxidant properties of CUR [8, 51]. Additionally, RES has been linked to reducing oxidative stress, inflammation, immune system function, substances produced from adipose tissue and the gut, as well as genetic predispositions that enhance the evolution of non-alcoholic steatohepatitis [52].

Tanase, Gosav [53] asserts that acute tubulointerstitial nephrotoxicity generated by xenobiotics, particularly cisplatin-related nephrotoxicity resulting from oxidative stress, correlates with elevated urinary levels of KIM-1. NGAL serves as a marker for acute renal injury and a dependable indirect indicator of oxidative stress, detectable in both serum and urine. Udupa and Prakash [54] asserts that biomarkers produced after tubular injury, such as KIM-1 and NGAL, can offer greater precision, earlier detection, real-time assessment, and proportionality to injury compared to conventional markers. In conjunction with [3], our data indicated that GS therapy significantly elevated tissue KIM-1 and NAGL levels.

Consistent with our findings, a prior investigation indicated that CUR mitigates cadmium-induced nephrotoxicity by decreasing urine levels of KIM-1 and NGAL [55]. Furthermore, CUR treatment significantly influenced the serum lipid profile, reducing the kidney and urine expression levels of KIM-1 and NGAL genes, while ameliorating oxidative toxic stress associated with diabetic nephropathy in rats [56]. Furthermore, CUR safeguards the kidneys from GS-induced renal injury by decreasing plasma concentrations of KIM-1 and NGAL [57]. Our findings demonstrated a significant enhancement with RES, which may corroborate the optimistic amelioration of kidney disease described by Copur, Yavuz [20].

The results of molecular docking showed that CUR and RES bind very tightly to AKT1 and DRP1. qPCR analysis verified this, showing that gene expression of AKT1 went up significantly in the GS-treated group. Both CUR and RES therapy brought gene expression levels back to normal. DRP1 serves as the principal regulator of mitochondrial morphology. DRP1 mRNA has significant expression in the human kidney, suggesting its vital role in the pathogenesis of mitochondrial-targeted renal damage [58].

A prior work demonstrated that a pharmacological inhibitor of DRP1 can mitigate mitochondrial fission during renal ischemia-reperfusion and cisplatin-induced nephrotoxicity [59]. Moreover, a prior investigation indicated that the protective effect of CUR against GS-induced nephrotoxicity may be partially facilitated by the preservation of mitochondrial functioning and biogenesis [60]. A recent study indicated that CUR exhibits nephroprotective effects against atrazine-induced damage by promoting mitochondrial autophagy and exhibiting anti-apoptotic characteristics [61]. After sepsis, curcumin enhances bioenergy in cardiac cells and mitochondrial dynamics through the sirtuin1-DRP1/peroxisome proliferator-activated receptor gamma coactivator 1-alpha pathway [62]. Moreover, as a potential risk factor for cardiovascular disease, RES is believed to increase mitochondrial biogenesis, mitophagy, respiration rate, and oxidation [63].

The activation of mitochondrial AKT1 signaling mitigated kidney harm from glomerulosclerosis during ischemia-reperfusion injury by reducing renal tubular damage [64, 65]. Aggregation of AKT1 in the mitochondrial matrix occurs upon activation, and the mitochondrion contains multiple identified substrates. Hexokinase II and glycogen synthesis kinase β are the components of the substrates, suggesting that AKT1 likely regulates mitochondrial functions [64]. In order to alleviate metabolic diseases and oxidative stress, CUR partly exerts its protective effects via controlling the phosphatidylinositol 3-kinases-AKT survival pathway [66]. In addition, reduced mitochondrial fission and autophagy are related with the nephroprotective action of CUR in maleate-induced kidney injury [67]. Researchers found that pretreatment with CUR reduced early-stage nephrectomy-induced changes in mitochondrial dynamics, bioenergetics, and oxidative stress, which may have a role in maintaining renal function [68]. New evidence suggests a link between mitochondrial dynamics malfunction and steatohepatitis, with RES potentially alleviating this condition [69, 70]. Through mitochondrial modulation via the AKT1/DRP1 pathway, the current findings suggest that RES and CUR actions may have promising nephroprotective benefits.

Histopathological examination of the GS group revealed marked structural alterations, including disorganization of glomerular capillary tufts, degeneration of tubular epithelial cells, and pronounced interstitial inflammatory infiltrates. These observations are consistent with previous reports describing aminoglycoside-induced tubular necrosis and glomerular injury. The predominant morphological changes included dilated renal tubules, in agreement with earlier studies [71, 72]. Additional tubular alterations comprised loss of the brush border in proximal tubules and thickening of the tubular basement membranes, corroborating findings reported by Volpini, Balbi [73]. The presence of proteinaceous casts further indicates tubular dysfunction. These lesions are attributed to the high affinity of proximal tubular epithelial cells for GS, which is internalized via megalin and cubilin-mediated endocytosis and accumulates in lysosomes. This accumulation triggers oxidative stress, lysosomal rupture, and apoptosis, ultimately contributing to structural and functional renal injury [46].

In addition to tubular injury, GS administration also induced notable glomerular alterations, including shrinkage, obliteration of capillary lumens, and mesangial hypercellularity, consistent with prior nephrotoxicity models [74]. Occasional thickening and irregularity of the glomerular basement membranes further indicated damage to the filtration apparatus. Focal inflammatory cell infiltration and interstitial congestion were also observed in some specimens, in agreement with previous reports [75]. Such changes support the notion that GS toxicity triggers inflammatory cascades, exacerbating tissue injury. These inflammatory responses are likely secondary to oxidative stress and cytokine release, which contribute to interstitial damage and apoptosis in renal tissues [76].

Although the precise molecular mechanisms by which CUR modulates apoptosis and inflammation are not fully elucidated, our findings support its protective effect on renal architecture. CUR-treated rats demonstrated marked improvements in both glomerular and tubular morphology, reflecting its ability to mitigate structural damage. Proposed mechanisms include stabilization of cell membranes by modulating their cation-binding properties, thereby reducing susceptibility to toxic insults [77]. CUR also suppresses the generation of reactive oxygen species and limits calcium influx, potentially through inhibition of sarco-endoplasmic reticulum Ca^2 +^ -ATPase and inositol trisphosphate-sensitive calcium channels [78]. Additionally, CUR may block cytosolic mediators that facilitate calcium entry, helping to prevent intracellular Ca^2 +^ overload [79]. By attenuating reactive oxygen species-mediated damage to the glomerular basement membrane, CUR may reduce proteinuria and preserve glomerular integrity [77]. The GS+RES group demonstrated the most preserved renal architecture, with markedly improved tissue integrity and minimal degenerative changes. In addition, RES has been shown to enhance mitochondrial function by promoting mitochondrial biogenesis and mitophagy, facilitating fatty acid metabolism, and reducing reactive oxygen species production [16].

Immunohistochemically, the GS-treated group exhibited reduced FOXO1 expression in tubular cells, while glomerular reactivity remained moderate, accompanied by a pronounced decrease in mTOR expression, particularly in tubules. FOXO1 is a key transcription factor regulating oxidative stress responses, apoptosis, and autophagy, whereas mTOR is central to cellular metabolism and regenerative processes [80, 81]. Increased production of MDA, decreased superoxide dismutase activity, and increased expression of collagen IV and fibronectin proteins in the renal cortex were all associated with FOXO1 downregulation [82]. Moreover, dysregulation of mTOR signaling disturbs renal cell homeostasis, leading to kidney disorders, including acute kidney injury [83]. In the current work, CUR and RES augmented the FOXO1 and mTOR pathways in GS-induced nephrotoxicity, as validated by robust binding affinity using molecular docking analysis. It was shown that the overexpression of FOXO1 results in less fibrosis development and suppresses inflammation in tubular cells through the antioxidant characteristics of FOXO1. Phosphatidylinositol 3-kinases/AKT/mTOR downregulation is observed in cisplatin-induced acute renal damage. Injuries to the kidney cause inflammation and cell death due to this pathway’s downregulation [84].

At the ultrastructural level, GS-treated kidneys exhibited disrupted mitochondria with sparse cristae, dilated endoplasmic reticulum, and nuclear irregularities, consistent with previously reported electron microscopic changes [74]. These alterations confirm mitochondrial involvement in GS-induced nephrotoxicity and underscore the central role of oxidative stress and impaired energy metabolism. Additionally, podocyte foot process effacement and cytoplasmic vacuolation indicate glomerular barrier compromise, which is associated with proteinuria and potential long-term glomerular dysfunction [85]. Transmission electron microscopy further confirmed the protective effects of CUR and RES, revealing well-preserved brush border microvilli, structurally intact mitochondria, and continuous glomerular basement membranes. Among the treated groups, RES exhibited the most preserved cellular architecture, with minimal degenerative changes and significant improvements in immunohistochemical markers. These findings underscore the potent protective role of RES against oxidative stress–induced renal injury.

Finally, CUR and RES treatment significantly reduces GS-induced kidney damage. By maintaining renal architecture, antioxidant levels, mitochondrial homeostasis, and the AKT/FOXO1/DRP1/mTOR signaling pathway, RES offered improved renoprotection compared to CUR’s moderate protection. The nephroprotective effects of CUR and RES were validated by histopathological, immunohistochemical, and ultrastructural examinations. These results validate the need for more clinical research into the therapeutic possibilities of RES and CUR as supplementary tactics to avoid GS-induced nephrotoxicity. The anti-inflammatory, antioxidant, and anti-apoptotic characteristics of natural compounds are drawing more attention as a possible means of reducing kidney damage in renal failure. Looking ahead, researchers are considering how to combine these natural compounds with traditional medicine and, if needed, nanotechnology.

## Data Availability

The datasets generated during and/or analyzed during the current study are available from the corresponding author on reasonable request.
